# The Crisis of Everyday Self-other Misalignments between Unaccompanied Minors and Adult Professionals: A Dialogical Self Study

**DOI:** 10.1007/s12124-025-09954-z

**Published:** 2025-11-26

**Authors:** Sarah Crafter, Evangelia Prokopiou, Nelli Stavropoulou

**Affiliations:** 1 The Open University, Milton Keynes, United Kingdom; 2 University of Northampton, Northampton, United Kingdom

**Keywords:** Unaccompanied minors, Dialogical self, Trust, Misrecognition, Professionals, Social care, Migration, Dialogical

## Abstract

Young people who travel across national borders as unaccompanied minors have become central figures in modern narrations about the ‘migration crisis’. At the macrosocial level, legal and policy frameworks intended to protect unaccompanied minors at times explicitly, or inadvertently, create everyday ‘crises’ at the microsocial level as part of asylum and welfare provision for children. In this paper, we use sociocultural approaches and dialogical theorising to examine how everyday ‘crisis’ are created at the psychological level, through misalignments in the self-other relationship between unaccompanied minors and professional adults. To do so, we add to these theoretical ideas the concepts of misrecognition and dialogical approaches to trust, to examine the forms of sense-making in both parties. We illustrate our theoretical points by drawing on data from the Children caring on the Move (CCoM) project. A dialogical analysis was undertaken on 112 semi-structured interviews with adults from several sectors (e.g. social worker, law, NGOs, healthcare, education, accommodation services, policy) and participatory approaches involving 75 interviews with 38 unaccompanied young people, including care object interviews and day-in-the-life interviews. The self-other misalignments discussed are ‘I-as-agentic-self’ as misaligned with ‘They-as-savvy-users-of-the-system’, and ‘I-as-detective/truth-seeker’ as misaligned with ‘They-are-testing-you’. We argue that both young people and adults can get caught in a dialogical loop of misrecognition, leading to the development of mistrust.

## Introduction

This paper is a contribution to this Special Issue examining sociocultural approaches to crisis and uncertainty. The aim of this paper is to use sociocultural approaches to dialogical theorising to examine the self-other dynamics between unaccompanied minors and adult professionals tasked with their care. Through the dialogical analysis examining ‘I-positions’ versus ‘they-other’ positions we illustrate how everyday crisis (both real and imagined) is ‘made’ at the level of ‘meaning-making’ by the young people and the adults through how they position each other. Their meaning making at the dialogical level is also influenced by wider macrosocial processes such as immigration regimes, frameworks and practices, as well as wider social representations such as migration-as-crisis narratives and child-as-crisis narratives. Seeped into macrosocial influences are generalised representations about child development and childhood, foregrounding often polemical perspectives on age and maturity which impacts on care practices (Crafter & Ott, online first). At the microsocial level, a dialogical conceptualising illuminates how both adults and unaccompanied minors put forward their own sense of ‘knowingness’, often dialogically contradictory, which contributes to the meaning-making of an unaccompanied minor a ‘crisis figure’.

To make this investigation, we draw on data from the Children Caring on the Move project, which aimed to investigate how unaccompanied minors, and those involved in their care (e.g. social workers, foster carers), make sense of, value, and take part in care relationships and caring practices within the immigration-welfare nexus in England. To aid us, we use three closely aligned theoretical approaches: (i) sociocultural psychological approach to dialogical self-other relationships, (ii) the concepts of recognition and misrecognition and (iii) dialogical approaches to trust and mistrust. In the context of this paper, a question arises around the sense-making between unaccompanied minors and the adults tasked with their care, as both knowers and producers of knowledge about their migration and navigation through asylum and welfare systems.

## A Crisis Made: Macrosocial Influences of Immigration Policy and Children’s Rights

Prior to 2010, the concept of crisis in relation to migration largely focused on small-scale or isolated events in individual countries. However, following the large movement of refugees and asylum-seekers in 2016, particularly from Syria, Afghanistan and parts of Africa, the notion of the ‘migration crisis’ as a global phenomenon gained prominence (Cantat et al., 2023). By the end of 2017 the number of refugees forcibly displaced by war or conflict was estimated to be 68.5 million, including 16.2 million newly displaced individuals. Children under the age of 18 years accounted for about half of the refugee population – an increase of 41% from 2009 (UNHRC, 2018). By 2023, around 40% of displaced people were children (UNHRC, 2024). Most recently, at the end of 2024, of the staggering 123.2 million forcibly displaced people, an estimated 49 million (40 per cent) are children below 18 years of age (UNHRC, 2025).

The relationship between crisis and migration is complex, as multiple factors can drive people to leave their countries, such as conflicts, environmental change or economic drivers (Cantat et al., 2023). Receiving countries also face challenges, and border governance can create crises that did not previously exist. Some scholars argue that migration should not be understood as a crisis but a mode of change with long historical ties (Heidbrink, 2014) or a series of ongoing, protracted crises (Mohamed Dafa & Fiddian-Qasmiyeh, 2023). Cantat et al. (2023) propose a framework for *Migration-as-Crisis*, which they describe as “the product of an assemblage of events, discourses/representations and practices” (p. 3). This perspective suggests that migration-as-crisis are both *objective*, reflecting real migration dynamics, and *subjective*, in that they are open to different forms of sense-making, socially constructed or interpreted differently, depending on the actor’s interests. The inclusion of children and young people provides another level of complexity because alongside narratives about ‘*migration-as-crisis*’ there are intersections with ‘*childhood-as-crisis*’ in the sense-making (Rosen et al., 2023). Scholars suggest the emergence of the ‘*child-as-crisis*’ figure came to the fore in 2016, during the same time as the ‘refugee crisis’, when a staggering number of young people began migrating without kin. The two became conflated (Lems et al., 2020). What emerged was an ambiguous figure who was initially represented as vulnerable because of their age and childhood status, and therefore in need of separate systems for humanitarian intervention (Heidbrink, 2020). However, they were treated ambivalently within immigration systems because of their immigration status (Allsopp & Chase, 2019; Allsopp et al., 2014). Legal frameworks such as the United Nations Convention on the Rights of the Child (UNCRC) sought to provide children like unaccompanied minors with the rights to legal protections regardless of asylum status and proposed that nation states should only act in the best interests of the child. Whilst this was positive as the basis of the legal framework, it heralded some unanticipated negative consequences for unaccompanied minors.

At the macrosocial dialogical level, many immigration systems are structured in ways that place different aspects on an individual’s identity in conflict. Unaccompanied minors, for instance, are required to prove their status as children through processes like age assessments, yet their immigration narratives are often met with disbelief during asylum processes (Wright, 2014). There are moral ambiguities and disputes over whether migrant children are “truly” children, a determination governed by the arbitrary age of 18 years. Emerging policies, care pathways, and practices contribute to the emergence of dual care systems that differentiate between citizen and migrant children (Derluyn, 2018).

To gain recognition as legitimate child migrants, unaccompanied minors seeking asylum are often expected to perform innocence and victimhood (Adams, 2009; Crawley, 2007, 2011). Protracted and complex asylum systems mean that children can wait extended periods for asylum decisions on their claims, during which some ‘age out’ before a decision is reached (Allsopp et al., 2014; Rosen, 2025). The psychological toll on young people is well-documented, not only due to difficulties in their migration journeys, but also because of the challenges associated with navigating a new culture, enduring challenging immigration and welfare systems, amid uncertainty about their future (Chase, 2019; Meloni, 2020). Further strains come from negotiating relationships with key adults such as social workers and foster carers (Allsopp & Chase, 2019; Crafter et al., 2021; Meloni & Humphris, 2019), and from the burden of public and media narratives that often portray them in a negative light (Rosen & Crafter, 2018).

In previous work, we have argued that alongside these powerful macrosocial elements, both unaccompanied minors and those professionals that work with them, are shaped by generalised representations of concepts of child development and children (Crafter & Ott, online first). In a qualitative meta-synthesis of 53 empirical studies involving adult professionals who work with unaccompanied minors, we found that adults often position these young people in polemical ways. In other words, when childhood is naturalized as a time of innocence and vulnerability (Burman, 2016; O’Dell et al., 2018), children whose lived experience differ from this ideal are frequently contrasted against it. Unaccompanied minors, for example, may be positioned as overly knowledgeable for their age, or, in extreme cases, as posing a risk or contamination to these imagined ideals. Some areas of developmental psychology maintain a clear demarcation between childhood and adulthood (Hendry & Kloep, 2010; Kessen, 1962). Within this framework, unaccompanied minors can be positioned as either “too behind” or conversely, “too mature” in comparison to their citizen peers. Children who experience diverse or non-normative childhoods (Lee, 2007; O’Dell et al., 2018; Woodhead, 1999), are ‘othered’ by physically or symbolically silencing and removing them from mainstream society(Crafter & Ott, online first).

### Microsocial Crises: Dialogical self-other Relationships Unaccompanied Minors and Adult Professionals

At the microsocial level, crisis can be understood as emerging within dialogical interactions between the self and the ‘other’. In the context of migration, this ‘other’ may take many forms: the voices of professionals, peers and other unaccompanied minors, institutions, or other individuals or groups within a young person’s social world. Asylum processes are, by their very nature, highly conflictual and therefore offer a valuable lens to examine self-other (mis)alignments (Tileagă, 2014). Dialogical approaches to self-other relationships are grounded in the understanding that human minds “do not function in isolation but are mutually connected”(Marcová & Linell, 2014, p. xvii). For instance, Hermans and Kempen (1998) argued that in the global-local nexus of increasing migration, the multicultural realities of the external world would compel dialogue between a multiplicity I-positions within the self.

The notion of I-positions emerged as part of the Dialogical Self Theory, with a basic premise that there exists ‘internal positions’ (e.g. ‘I’ as a unaccompanied minor, ‘I’ as a child), ‘external positions’ (e.g. the imagined voice of my social worker, peer, foster carer) and ‘outside’ positions (people or groups in the outside world)(Hermans & Gieser, 2012). Others scholars have proposed that the multiplicity of identities in multicultural societies can lead to a ‘collision of viewpoints on the world’ through sociolinguistic hybrids (Bakhtin, 1981, p. 360), opening up a ‘third space’ in the construction of a hybrid self (Bhatia & Ram, 2004) and in the case of young people in less powerful positions, dominant identities imposed upon them (van Meijl, 2012).

Bhatia (2012) argues that a dialogical model of self helps us analyse how I-positions are both constructed and constrained. In her study of the Indian diaspora in America, she suggests that migrants are frequently “being positioned” or “being placed” by others (p. 123). For unaccompanied minors similar dynamics arise: their very right to exist, the migration stories they tell, and their stated ages are questioned by more powerful adults around them (Allsopp & Chase, 2019; Allsopp et al., 2014; De Graeve & Bex, 2017; Wernesjö, 2021). Drawing on Mead, Vygotsky and Bakhtin, ‘dialogue’ extends beyond face-to-face interactions. It also “echoes the voices of discourses that were held elsewhere at other times and in other situations (Grossen & Salazar Orvig, 2011, p. 492). Marková (2006) provides a dialogical framework for this understanding, by drawing on Salgado’s concept of ‘other-in-the-self’ (Salgado & Ferreira, 2004) to propose two related constructs: *External Alter* and *Inner Alter*. The External Alter refers to the action of speaking to ‘real’ people whilst the Inner Alter is engaging in an internal dialogue with a number of less visible, symbolic ‘others’. This perspective invites critical reflection on how key parties, such as unaccompanied minors and the professional adults who work with them, make sense of what they know and what is known about each other. What happens if there is a misalignment in the self-other relationship? How can we theoretically understand this misalignment as a crisis?

In their study with unaccompanied minors in Finland, Korkiamäki and Gilligan (2020)suggest that a central challenge these young people face is the everyday experience of misrecognition. Drawing on the foundational work of Honneth (Honneth, 1995a, b; Taylor, (1994) and Fraser (2000, 2020), they describe how unaccompanied minors are perceived differently because of their refugee status, which some young people accept as part of their identity and others reject. Korkiamäki and Gilligan (2020) argue that unaccompanied minors are continually subjected to discourses of misrecognition as part of their everyday lives. In response, they may internalise these narratives, resist through acts of agency, conform by presenting themselves as a “good human being” (p. 6) or seek acceptance through displays of “ordinariness” (p. 6). According to Taylor, identity is “partly shaped by recognition or its absence (Taylor, 2009, p. 25) and Honneth’s three axes of the self (i) Self confidence (in relationships), (ii) self respect (through recognition of legal and moral rights) and (iii) self-esteem (through solidarity with a community or group), speak to the deeply psychological features of these self-other identity processes.

For unaccompanied minors, processes of (mis)recognition can be best understood as a dialogical interplay with power and agency (Amer & Obradovic, 2022). Asserting or resisting claims for power and agency are particularly difficult for unaccompanied minors who are questioned because of their child status and judged on their deservingness because of their migration status (Leon & Rosen, 2023; Wernesjö, 2021). We draw on a relational approach to agency, which does not view agency as essentialised within the individual (Raithelhuber, 2016). Rather, young people’s capacity for agency is “socially, relationally and materially constituted, rather than being an essential quality or production of the individual child” (Callaghan et al., 2025, p. 8). The notion of a capacity for agency can be extended to some of the professional adults who work with unaccompanied minors, who have varying room to manoeuvre when they operate within the boundaries of certain policy and practice regimes, roles and responsibilities (Crafter et al., 2021).

The other important element in the self-other dialogical interplay between unaccompanied minors and those professionals tasked with their care, is trust. Perceiving oneself as misrecognised by the other represents a step towards the erosion of trust. Misalignments between what is known and who is recognised as the ‘knower’ within self-other relationships, can be understood as acts of mistrust (Linell & Keselman, 2012). Recognising that trust and mistrust are multifaceted and context-dependent, Marková et al. (2008) provides an in-depth model of trust as a quadrant with four dimensions: (a) primary (or basic taken-for-granted) trust at the micro-social which refers to innate sociability and openness to others; (b) reflective trust at the micro-social level, focusing on the Inner Alter dialogues and an inner awareness of trust; (c) generalised trust at the macro-social level, linked to others in society and (d) context-dependent trust, as the macro-social dimension which signifies context specific forms of trust in the manner of “complex social phenomenon-or we could say, a complex social representation-appearing in different guises (p. 19). The final form might include generalised representations of concepts of children’s development and childhood as discussed above.

Previous research examining expressions of trust or mistrust have usually done so using recorded conversation between two specific parties. Tileagă (2014) makes the point that expressions of trust and mistrust can be expressed in subtle or abstract ways. In conversation, interlocutors might not always explicitly express their mistrust in their discourse. It would be unusual for a young person to tell an adult in a position of power ‘I don’t trust you’. Linell and Kesleman (2012) in their analysis of conversations with Russian young asylum-seekers in Sweden and Swedish Immigration Officials, were able to show how even though mistrust can be difficult to identify in discourse, misalignments between what is said by one party and what is received by the other, are a key signifer. They noted that there were escalations in expressions of mistrust during official interviews that made up a series of mistrust sequences.

In the relationship between children and young people and the adults who interact with them, trust operates on both interpersonal and epistemic levels (Zittoun, 2014). Interpersonal trust is particularly challenging in contexts where one party holds a greater position of privilege and power; such as a social worker in relation to the young people they support. Epistemic trust refers to mutual trust in shared forms of knowledge, interactions and joint activities (Marková, 2025). Zittoun (2014) writing about epistemic trust in the context of education, describes the teacher as a mediator between the *object of knowledge* (what is to be learned) and the *learner* (who is the child). A students’ commitment to the object of learning depends on their trust in the teacher’s ability to convey that knowledge effectively. For unaccompanied minors, however, epistemic trust presents two major challenges. First, the object of knowledge is diffuse and across multiple domains - welfare, education, health provisions, the asylum system and managing their case, navigating a new culture and the everyday world, accessing the right services for those provisions, and understanding their own asylum rights. Second, unlike the student-teacher dyad, the immigration and welfare system means that each of these objects of knowledge is overseen or understood by a myriad of different professions and professionals.

The research literature on unaccompanied minors provides numerous examples that suggest both interpersonal and epistemic trust are implicated in professional and institutional relationships. Long-standing austerity measures, continual organisational restructuring, and reduced staffing capacity have contributed to an erosion of trust in social workers, particularly when there are demands from immigration officials to share information about young people (Hadwin & Singh, 2025). In their discursive analysis of the dialogical exchanges between asylum-seeking young people and migration officials during asylum assessments in Sweden, Aronsson and Osvaldsson (2014) describe interactions of “veiled morality” or “veiled stances” (p. 45). These interactions involve unfolding discussions in which “blame-implicative narratives” emerge through a series of contestations about the stories told by the young people.

It is important to note that, while there are strong examples of conflict emerging through direct talk or interactions (Grossen & Salazar Orvig, 2014; Linell & Keselman, 2012), everyday descriptions of trust and mistrust can remain abstract. As Tileagă (2014) observes, such abstractions “feed deep into our ways of talking about personal and public experiences, people, and social relationships” (p. 55).

## Methodology

The project discussed in this paper sought to investigate how unaccompanied minors, and those involved in their care, make sense of, value, and take part in care relationships and caring practices within the immigration-welfare nexus in England. In particular, we wanted to understand unaccompanied minors’ experiences of care, and caring for others. The study, called the Children Caring on the Move project (CCoM), ran between 2019 and 2023 and was funded by the Economic and Social Research Council (ES/S001980/1). This paper draws on interview data with two main groups (i) unaccompanied minors living in two large towns in England and (ii) a range of adults who work directly with young people, as well as adults within procurement, accommodation and policy roles.

## Data Collection with Unaccompanied Minors

The interviews with unaccompanied minors were undertaken as part of a participatory action research approach. A group of Young Researchers with migration experience were trained as co-researchers by members of the academic team. The academic team were formed of researchers from University College London and the University of Northampton. The Young Researchers helped to refine the research questions, design the methods, undertake interviews, engaged in analysis and supported dissemination(Aisha et al., 2024). Interviews with unaccompanied minors were jointly conducted by a Young Researcher and one academic member of the team. There were 75 interviews with 38 unaccompanied minors from two major cities in England. Each young person was invited to 2–3 interviews over a 6–12 month period. These included (i) object-based interviews where participants were asked to bring an object that represents care; (ii) photo voice focused on a ‘day in the life’ of the participant; and (iii) walking interviews to see places of (un)caring. For ethical reasons, details about the interviewees remain very light because at the time of data collection, many of the young people who took part in the study were undergoing extremely protracted asylum claims processes, which could potentially still be ongoing.

## Data Collection with Practitioner and Professional Adults

Data collected with adult practitioners and professionals involved semi-structured interviews with a total of 112 adults. The design of the interview schedules and the interviews themselves were carried out by researchers from The Open University, University College London, University of Bedfordshire and University of Oxford. The first trench of data was collected in the geographical areas of two anonymous Local Authorities in England, selected because they are home to relatively large numbers of unaccompanied minors. There were 63 adults who were interviewed because of their roles and responsibilities linked directly with the care of unaccompanied minors and covering most of the key sectors such as social care and education (see Table 1. for a detailed breakdown). Following on from initial conversations with the unaccompanied minors, a further set of interviews were undertaken with unregulated accommodation providers such as company directors, procurement staff and frontline workers. Finally, a further 11 interviews took place with regional and national-level policy makers and professionals about broader policy issues. Across all three trenches of interviews, some took place individually with a minority being interviewed in pairs.

**Table 1:** Overview summary of the adult interviews

Interviewees	Sector(s)	Interview schedule themes
Frontline professionals and practitioners working with unaccompanied minors	Social care (*N = 15*), education (*N = 6*), health (*N = 3*), mental health (*N = 2*), border force (*N = 2*), charity/NGO’s (*N = 22*), and interpreters (*N = 1*), Immigration lawyer (*N= 5*), foster carer (*N = 5*), Support worker in accommodation (*N = 2*)	Interviewee’s broad experience of caring for unaccompanied minors and their role in their lives; the interviewee’s own understandings of care, care relationships and caring practices; how care changes over time; their views on the wider economic, social and political priorities and challenges that influences their ‘care’ and support practices
Irregular accommodation providers	Company directors (*N = 15*) Local Authority procurement and placement staff (*N = 10*); frontline Local Authority workers (*N = 10*); and third sector advocates (*N = 3*)	How accommodation is provided and details about provision; ownership details for the property; how the interviewee became involved in accommodation provision; details about the business modal; how the accommodation was established; the financial challenges in relationship with the Local Authority.
Regional and National policy representatives	CEO of a Children’s Trust; Police role in Modern Slavery and Organized Immigration Crime Unit; Senior Social Work Consultant; Head of a Fostering Agency; Local Government Association: Policy and Finance; Education Policy Partnership; Unaccompanied Asylum-Seeking Regional Local Authority coordinator; Consortium commissioner; Local Authority intake representative; Senior Legal and Policy Officer	Role within the organization; how the role relates unaccompanied minors; perceived most pressing issues; work most proud of; key challenges faced; impact of wider welfare rules and immigration policies; how care is understood

## Ethics

We received ethical clearance from Open University (HREC/3219/Crafter) which was reviewed and supported by University College London. In keeping with the ethical considerations central to participatory action research, we engaged in ongoing ethical reflections for the full duration of the research process. At no point did we attempt to connect the adults we interviewed with the young people. We deliberately did not seek to recruit young people via any State-related institution or organisation. The study began just before the start of the Covid-19 pandemic. Some of the Young Researchers had begun their participatory training in-person. This was paused in the initial uncertainty of the pandemic whilst informal contact was maintained at the request of the young researchers, to stay connected with support during a difficult time. Four months later the training resumed online. Some of the interviews with unaccompanied minors was conducted online and then when lockdowns ceased, they resumed in-person, as originally planned. All of the adult interviews were undertaken online.

### Research Team and Analytic Process

We had two teams of researchers working on the data collection with young people and adults respectively, and from a range of disciplinary backgrounds. The team working with the Young Researchers and unaccompanied minors included three Co-Investigators (including Author 2), one Post-Doctoral Research Assistant and one Research Assistant. The team brought a range of expertise including work with young people, unaccompanied minors, participatory research action, intersections of race, gender and migration, psychological approaches to dialogical identities and frontline charitable work. The team interviewing the adults included the Principal Investigator (Author 1), two Co-Investigators and a Post-Doctoral Research Assistant with combined experience in psychological approaches to children’s migration, foster care and social work practice, and political economic of care/care chain expertise. The third Author was brought into the project to provide support at the analytic stage, drawing on their expertise with child and adult refugee studies. The overall study was supported by an Advisory Group with both academic and professional experience from a range of sectors.

**Fig. 1:**
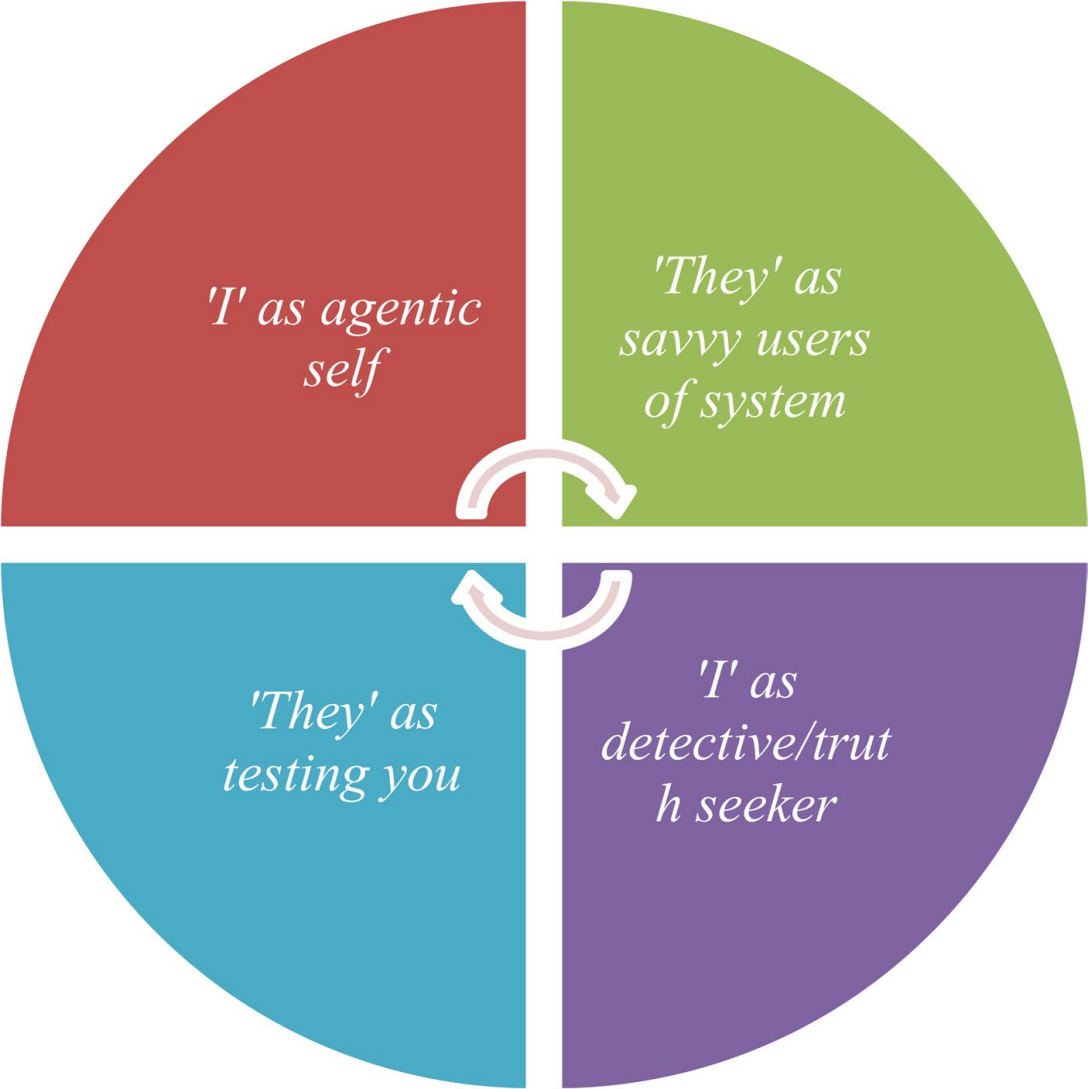
Interconnected ‘I’ to ‘they-other’ dialogical positions

The data were coded according to dialogical positions that cover (1) I-positions, (2) They-other positions, (3) We positions and (4) social representations(adapted from Mahendran, 2018). From the array of different dialogical positions, the positions identified in Fig. 1 were chosen to illustrate how the self-other dynamics create everyday ‘crisis’ at the level of meaning-making. Specifically, this paper draws on two sets of interrelated self-other positions: ‘I-as-agentic-self’ as misaligned with ‘They-as-savvy-users-of-the-system’, and ‘I-as-detective/truth-seeker’ as misaligned with ‘They-are-testing-you’.

## Findings

As discussed in the introduction, the concept of crisis is not new in research on the migration of unaccompanied minors, or children more broadly (Heidbrink, 2020; Lems et al., 2020). Our adult respondents often invoked the term ‘crisis’ within the context of their interviews, and their examples of what counts as a crisis reflect some of the key findings from the wider literature. Several participants described how systemic crises impacted on their professional practice. Bella, a paediatrician, talked about how the rise of a “*global asylum crisis*” after 2016 made her realise she lacked the skills to assess the complex needs of unaccompanied minors. Bruce, who worked for an independent foster agency, and Ethan, working in accommodation procurement, both talked about an “*acute crisis*” (Ethan’s words) in foster care. They described severe shortages of foster placements willing to accept unaccompanied minors, with competition between providers driving-up high-cost foster placements to Local Authorities. Lauren, a Project Manager in an Arts charity, and Charlie, who also worked in a support charity, described a convergence of crises, such as mental health crisis, housing crisis and health crisis, compounding each other.

Others referred to *crisis* in a more personal way, often related to the very real challenges associated with mental health, trauma and the effects of difficult migration journeys. For example, Bailey, a paediatric registrar, described the challenges of working with young people who arrive “*in a state of crisis*,” a condition she characterised as all-consuming. Nova, who worked for an accommodation provider, framed crises as a normal part of the young people’s everyday lives. This sense of a *normalised crisis* was echoed by other adults in our sample, particularly in relation to mental health, where barriers to accessing support are so entrenched that crisis has become the norm. Only one young person, Zoran, used the word ‘crisis’ in their interview, and then, only when referring to reading the term in a newspaper article. Zoran noted that ‘crisis’ sat alongside other negative words such as “fears”, “attacks and fights”.

Overall, these broad uses of the word *crisis* by the interviewee’s suggest that it is understood as something borne within the individual, through descriptions of individual mental health crisis, or are the product of a confluence of systemic failures, many of which have a long and protracted history in the UK context. Nadia, a project manager in a charity argued that as long as young people remain without an accepted asylum status, they live in a permanent state of crisis because “*It’s not possible for them to kind of move through that natural progression to independence*”. She described how the young people can remain in a form of developmental liminality, being potentially 24 years of age yet still requiring high levels of care. Nadia’s point about young people being ‘stuck’ in the child-like position, together with Brent’s remark that “*young people are in a crisis anyway*,* nationally*” (Brent, accommodation provider) speaks to the close intersection between representations of childhood and migration.

To this point, such perspectives on crisis within unaccompanied minors’ migration experience are well-documented in the literature. However, through the dialogical analysis examining ‘I-positions’ versus ‘they-other’ positions we illustrate how the crisis is also ‘made’ at the level of ‘sense-making’ by the young people and the adults through how they position each other. Each puts forward their own sense of ‘knowingness’, often dialogically contradictory, which creates around the young person a ‘crisis figure’.

## ‘I’-As-Agentic-Self Misaligned with ‘They’ as Savvy Users of the System

### ‘I’-As-Agentic-Self

Findings suggested that misalignments in self-other relationships arise because young people - who have often, out of necessity, become highly agentic in advocating for themselves within a challenging immigration and welfare system - are negatively positioned by adults as savvy manipulators of that system. We begin by presenting the unfolding **I-as-agentic-self** position held by some of the young people in the sample. It is very likely that the unaccompanied minors had exercised their capacity for agency many times before, and during, their migration journey to England. In this paper, we pick up their narratives during their journey through the asylum and welfare systems following their arrival to England. This first example, from Star, provides a useful starting point for how or why young people might need exercise their capacity for agency within this complex asylum and welfare system:

- *They don’t really … they don’t really give you directions*,* they don’t tell you what to do*,* they don’t tell you where to go when you need help. You just have to figure it out by yourself*,* or you just have to ask people around and some people are not really willing to share things*,* so it is really hard*,* yes….(Star*,* Unaccompanied Minor)*.

Star’s account suggests that, for her, when she needed help and direction, the key adults around her failed to provide the information she required. This was not an uncommon among the young people interviewed in the CCoM study. Many identified systemic as the main reasons for developing skills becoming highly self-directed in seeking-out information, resources and support.

It also become evident through both the professionals’ and young people’s narratives, that developing capacities for the agentic self, against a backdrop of complex relational and systemic challenges, is a long process. For example, Salim (unaccompanied minor) described how his initial relationships with various social workers seemed positive. They would ask about his life and interests, such as his favourite sports, which helped to build rapport. As Brandon, a social worker explained, they like to “*Get alongside the young person*,* find out what they’re interested in…*”. This was often seen as a key relationship-building exercise, intended to foster trust while also helping to build a detailed case record. As Brandon went on to say:

- *But I mean*,* all the disclosures you’ll get*,* you know*,* and serious disclosures of sexual abuse and stuff like that*,* you never get them in a dining room table*,* it’s often when you’re sat next to each other on a bus or in a car*,* or somewhere where they’re completely distracted by something else….” (Brandon*,* Senior Social Worker)*.

From the young people’s perspective rapport-oriented questions were initially well received. However, over time, and in the absence of practical “*direction*” as Star puts it, contributed to young people feeling that this approach was not accompanied by meaningful action. This in turn contributed to some of the young people increasingly exercising their capacities as **‘I’-as-agentic-self**.

For Salim, he had experienced three foster placements and five different social workers, with the quality of those relationships varying considerably. During his interview, he described one particularly positive relationship with a social worker, who later became over-burdened with clients. Overall though, Salim felt he was just one client among many, which led to any requests around housing and asylum being drawn-out and frustrating: “*Obviously the social services they don’t care. The social workers*,* especially the social worker because you are not the only case that he has.*” To compensate, he navigated his own path as ‘I’-as-agentic-self, through the system:

- *I’m feeling very good*,* you know. Full of experience. Now no one can do anything to me like*,* they can’t. Like before they were promising me*,* I give you this*,* this*,* this*,* that. They will show me everything like in my dream. But they done nothing to [for] me. They’re giving nothing*,* you had just – they’re just playing with me*,* with my mind. I was happy all they will give again. I spoke this*,* this*,* this. Only the second time they came to me they forgot everything. I said*,* “No*,* that’s not work. If you promise to give me a lot of*,* give me by end of this month. Look*,* four weeks you can do anything” (Salim*,* Unaccompanied minor)*.

Salim explained that social workers would offer to help him, when he followed-up with specific requests, they frequently told him they were too busy. Promises of concrete actions would routinely take months to materialise. His account speaks to a sense of repetition and frustration, when he says, ‘*I spoke this*,* this this’.* It may refer to the recurring questions aimed at building asylum case, as Brandon mentions above. However, Salim’s narrative suggests that, for him, epistemic trust has been broken because the shared understanding attached to ‘*this*,* this*,* this’* are not realised. It could be possible that the professionals Salim encountered were overwhelmed with bureaucracy. Yet, as Marková et al. (2008) note, such procedural delays can also be symptomatic of a broader erosion of trust in professional relationships. Either way, Salim was left feeling that he was caught in a ‘*game*’ whilst ‘*they*’ were playing with his mind. His response was to move to a more agentic approach to his own asylum journey where he became “*full of experience*”. The quality of ‘full experience’ arguably runs counter to generalised narratives of childhood which tends to position ‘experience’ as a slowly developing cup that is filled in the act of ‘becoming’.

The multiplicity of encounters with adult professionals and practitioners meant that some young people were able to find pockets of active support. In this next quote Dozan describes the mistrust he had for some professionals and the avenues of trust and support he garnered in other spaces:

- *Yeah*,* but if you want my opinion*,* never believe what they say*,* from social services*,* from your solicitor as well*,* from your support worker as well. But there’s organisations*,* like [advocate]*,* or*,* like*,* this project that we’re doing*,* and that…yeah*,* because [advocate] was so helpful for me*,* and because of you guys*,* I learned a lot as well. If I have*,* like*,* if I was having*,* like*,* at this point I’m having now*,* I was never not going to take that long*,* I was going to get my status*,* without even a solicitor*,* without even asking people to find a solicitor. But I learned that because I was going to Red Cross*,* like*,* having this project with you guys*,* and a lot of other things. I get*,* like*,* more information what to do*,* and that’s what is the best thing*,* like*,* to join a group like that*,* or projects like the research*,* to get to know the rules and the system and the things clear*,* to be clear for you (Dozan*,* Unaccompanied Minor)*.

The need to enact an ‘I’-as-agentic-self’ position in the welfare and asylum journey is described in this next quote as a necessary act of courage. When asked what advice he would give to other unaccompanied minors arriving in the UK, Zoran replied:

- *Ah*,* okay. I’m saying to them [other unaccompanied minors]*,* it’s…don’t be scared or afraid for asking to help*,* asking to get support*,* because I did lots*,* I tried so hard to find charities*,* organisations*,* helping*,* friends*,* no-one is knocking at your door to ask you that you need support or something. This is just*,* like*,* the people which is…I think it’s less and it’s different for some situations. But you have to do it by yourself*,* you have to ask and ask*,* you have to email*,* you have to phone*,* you have to get support by yourself*,* just yourself*,* because no-one is doing that for you*,* no-one is caring about what you’re doing*,* what’s your plans*,* because that’s always yourself. If you just sit and wait until the Home Office give you a letter or to send a letter*,* you are going to sit a skeleton… Zoran (Unaccompanied minor)*.

Valsiner and Cabell (2012) talk about the dynamics of dialogue within the self-system and argue that some I-positions are promoted and others are suppressed or constrained. The lethargy in the system led Zoran to achieve an I-position as an active doer - ‘*I did lots*’ and ‘*I tried so hard*’. Overall, the path to the agentic self is borne out of a necessity to manage a complex system and challenging relationships.

It is important to note that not all of the young people narrated a position of ‘I’-as-agentic-self. The capacity to exercise agency is dependent on the relational, social, material and power structures at play in any given time or circumstances. Waithood remains a central feature of the asylum process for unaccompanied minors, many of whom endure prolonged previous of uncertainty as their claims and appeals progress over years. In that time, Rosen (2025) describes that vernaculars of waithood include feeling “stuck” “crazy” and “drained” (p. 7), though the care of friends and community can offer an important antidote.

In relation to self-other relationships with some professional adults, the consequences of waithood manifest as deep misalignments in trust and understanding:

- *They break us so much emotional and the physical. They’re playing. They’re playing a game. Joshua (unaccompanied minor)*.

Here, Joshua frames the self-other relationship as a ‘game’ where he takes an I-as-a-puppet position which would make the ‘other’ adults in the system, the puppet masters. Joshua described a variety of situations as being like a “*prison*”. Joshua felt physically restricted in terms of his accommodation and his ability to work or visit friends, as he says *“…And this is like to stay in a home*,* in a house like a prison. And for me the prison is like*,* you know*,* you’re not going out*,* you’re not allowed to work*,* and you don’t have like allowed to study and stay at home. That’s a prison for me*”. Equally he is subject to the vagaries of sudden decisions to move him where the authorities would like “*So what can I do? Nothing. So I have to just*,* you know? They say to me*,* “Move this place*,*” I move. “Get this thing*,*” I have to get. Yeah*”.

The capacity to enact an agentic self could also be challenging for some of the professionals we interviewed. Tasneem, a support worker with a charity, was asked if there was anything in her role she wished she had done differently. She replied:

- *I think I wish I had been less… what’s the word? I wish I’d been more assertive from day one… And it’s one of the things that goes back to saying what’s made our work so difficult and it’s that; it’s the mind-games that we’re constantly playing and having to navigate this minefield where I could step on something and it could be explosive because I’ve trusted somebody. So*,* I think that I would’ve been assertive from day one. And now that I am very assertive it means that people have upped the ante on personalising their attacks against me but that’s okay. I think that you have to be like that because at the end of the day it is the only way that you can best protect these young people and*,* like I said*,* I have seen institutional abuse*,* like I have seen people in positions that are supposed to care for these young people in positions of power really abuse that power and fully traumatise young people. (Tasneem*,* support worker for a charity)*

Challenges in trust and mistrust do not just occur between the adults and the unaccompanied minors, but also between the groups of multi-professional teams. Each professional brings their viewpoints, roles, practices, and personal values to bear on any given case. Hadwin and Singh (2025) report that in the small sample of young people in their study, 20% of workers were considered hostile and 80% were supportive. For Tasneem, situations where trust was broken led to her becoming more “*assertive*”. Like Salim, she invokes a sense of being manipulated with “*mind-games*” where the stakes are “*explosive*” if trust is wrongly placed.

### ‘They’ as Savvy Users of the System

Whilst some of the young people positioned themselves as ‘I’-as-agentic-self out of necessity, we argue in this paper that this gives rise to a form of misrecognition in the form of ‘they-other’ position by adults, as **savvy users of the system**. It is at this level of meaning-making where a dialogical crisis is created. Across our sample, we had adults positioning young people as highly competent ‘knowers’ of the system and at times, a threat to the system. This was questioned by Tess, an immigration lawyer, who outlined a generalised view of unaccompanied minors that permeates through the systems and professional practice, and is also reported in the wider academic literature (Chase et al., 2020).

- *So*,* the politics of it all – I mean I read a judge’s decision recently*,* where he said that these young people know that if they claim to be under 18 they go into the care system*,* which again somebody coming from [Name of place] having travelled from Afghanistan probably knows little about the care system. Any more than they know what asylum is – they don’t know! So*,* they are having to deal with these assumptions about asylum seekers*,* assumptions about migrants*,* assumptions about people from certain countries*,* assumptions about young Muslim men*,* as a lot of them might be. Tess (Immigration Lawyer).*

Tess’s role within the asylum system, as a lawyer who collates asylum narratives from unaccompanied minors, provided her with valuable insight into the ways young people are often assigned ‘placed’ dialogical identities as savvy knowers of the system. Her position enabled her to challenge these assumptions. However, direct engagement with young people alone does not necessarily foster a critical approach to self-other positioning, as adults themselves are subject to both macrosocial forces and microsocial influences in their own roles and responsibilities (Crafter et al., 2021).

In this complex discussion by Tannish, a social worker in a managerial role at a Local Authority, he shifts between a position of ‘They-as-aspirational’ through to ‘They-as-savvy-users-of-the-system’. In his account, Tannish initially positions young people as being in the UK for aspirational reasons – to have a better future. However, his dialogue then turns to their perceived responsibilities toward family members, suggesting that their silence about pre-existing family connections in the UK functions as a mechanism by which to cheat the system:

- *Very simplistically you see, actually they are just getting their head down, because that is what they want do to – they just want to get their head down, they know what is important to them, they are absolutely aspirational and yet – but, the other context again from my experience and everything else as well, they are still thinking of a family or significant others from wherever they come from… the vast majority in the context of why they are here, and the context is not just about me, it is about me and my bigger family, and they will share who their bigger family is when they ready to share who their bigger family is. And, that bigger family is very much dependent on the immigration status, and we have just accepted that this is just life and this is what happens. So, when they do get a positive decision, they do get a positive decision and young people are then able to – we use that language – they are able to actually share details of potentially a brother or a cousin or another relative that actually lives in [name of city]…But, that information wasn’t forthcoming beforehand, because they know full well that if they would have shared that, that might have an impact on their immigration, because that might actually mean well actually you have actually been supporting an economic migrant, aren’t you? You are actually not fleeing, you have been financially supported to arrive in this country, and we take a very, very neutral view – you know what, this is just what life is and this is what happens, as well. (Tannish, Senior Social Worker)*

Being aspirational is positioned by adults as a positive identity, although in previous work we and others have argued it is often contingent on young people presenting themselves in ways that aligns to social workers and ultimately, financial support (Crafter & Ott, online first; Devenney, 2020; Meloni & Humphris, 2019). It is noteworthy that Tannish conflates being an ‘economic migrant’ and ‘not fleeing’ a country. Even when young people do have family connections in the UK, those relatives may be unable, or unwilling, to provide support. In addition, the existence of a family connection does not negate the reasons for the push to leave a home country, such as fleeing war or conflict. Although family-related information might have had an impact on their claim, it would in theory work in their favour. Article 8 of the Dublin III Regulations^1^ specifically supports family reunification in another member state. Whilst Tannish claims to take a “*neutral view*,” the self-other position is treated as one of suspicion.

## ‘I’ as Detective/Truth Seeker Misaligned with ‘They’ are Testing You

### I as Detective/Truth Seeker

In this section, we examine forms of self-other misrecognition through the lens of **‘I’-as-the-detective/truth seeker**, as articulated in some adult dialogues, and how this is recognised by unaccompanied minors who are aware of being tested within the system. Whilst developing an understanding of the young person is key to many of the relationships between young people and adults (e.g. teachers, lawyers), social workers are uniquely required to know as much as they can about the young people in their care (Ward, 2022). The reasons are two-fold. First, under statutory guidance the social worker must engage in “triple planning” as part of the ‘durable solutions’ agenda for their future i) a future in the UK if they are granted long-term permission to stay ii) a return to their country at some point in the asylum process and iii) an interim or transitional plan to focus on short-term goals whilst status remains uncertain (Hadwin, 2022). Second, the information gathered contributes to the young person’s asylum claim, which often takes placed within a wider climate of distrust aimed at ‘weeding-out’ genuine children from those posing as children (Eastmond & Ascher, 2011).

Brandon, a senior social worker for the state, discussed the importance of stringently following the guidelines for assessing unaccompanied minors’ legitimacy. In the UK, the Merton Guidelines are a set of principles for assessing the age of young asylum seekers. The principles speak to ensuring a fair and holistic assessment and taking a child-centred approach. As part of those guidelines there must be two social workers and an interpreter present if needed, during the assessment. There also needs to be an appropriate adult “*like you would do in custody at a police station*,* just to make sure the process is fair*” (Brandon). Whilst Brandon frames this as an issue of fairness, the reference to a custody situation sets-up a combative ‘they-other’ dynamic.

He went on to explain how social workers “*try to build a chronology of that young person’s life*,* to go through their health*,* education*,* their life experiences before coming to the UK*,* family history*,* the journey they’ve taken to the UK*,* independent skills*,* hobbies.*” All areas of life a mined for information including the views of other ‘trusted’ figures such as “*foster carers or keyworkers*,* the school*,* the allocated social worker*,* and any other professionals who may interact with that young person.*” Continuing the police analogy introduced by Brandon, and reminiscent of the detective gathering evidence, assessing social workers may also draw on “*intelligence from the Home Office*” including fingerprint data from pre-arrival to the country. He goes on to say, “*And the cherry on the cake usually is having social media evidence*,* because that’s a very strong*.” Brandon recognised this as a “*controversial area of social work*” but framed the outcome as a safeguarding measure that protects the genuinely vulnerable child across foster care placements, schools and the community.

As part of this extensive information-gathering process, every aspect of young people’s lives are examined and so is the community in which they are situated. In this next quote, Tannish, who is a social worker by background, describes the suspicions he and his team sometimes hold around the young people’s hobbies and activities. Engaging in out-of-school clubs and sports is usually framed as positive for skills-building in the transition to adulthood for citizen children. In this case they are reframed in a negative light for unaccompanied minors:

- *We also then have to be – at another level we also have to be mindful of the community, as well. The community is there to be supportive, but the community also can actually be a point of vulnerability and exploitation of these same, young people. And, then we talk about kind of – some of the staff [his social work team] would have said so, your barbers and your car washers and your hospitality trade and why is it you go to some of these Indian weddings and all the young people are there, all dressed up, what is going on here? It is that sort of conversation, it is that sort of conversation. You also then need to be mindful of examples of [name of park], and all these young lads – because 90% of the asylum-seeking people are male, that is just a reality – so, when I say lads that is what they are, and they are all kind of playing cricket in the middle of winter, and as a former cricketer you think oh wow, how fantastic is that! … then you start gathering the intelligence that is what is really then more hideous is there is a game of cricket going on, and there is betting going on at the side, and it is actually some of the players are being coercively controlled through matches, and then [name of person] or someone told me that, and I was like you are joking! That is exactly what is happening! Tannish (Social worker, state).*

Young people’s vulnerability to exploitation should not be underestimated or overlooked. However, migration scholars have argued that such vulnerability often stems from the structural conditions of the asylum system itself. For instance, the inability to seek lawful employment, which can push young people into precarious or risk situations (Crawley & Skleparis, 2018; Digidiki & Bhabha, 2018). Playing cricket in the park could be positioned as idealised childhood activity, yet under the gaze of suspicion, even this can be recast as a potential side of criminality or crisis.

Suspicion is also placed upon the wider community, revealing a systemic lack of trust beyond the young people themselves. It is unclear what meaning Tannish intended when he remarked “*so*,* when I say lads that is what they are*,” but this could allude either to the misogynistic, pack-like behaviour associated with ‘lad culture’ (Owen, 2020) or be a reference to their youthful potential for exploitation. The link to being “*coercively controlled*” suggests it is the latter. Interestingly, like Brandon, Tannish invokes the notion of “*intelligence*” gathering which places him in the dialogical position as that of someone uncovering truths much like a detective.

#### ‘They’ are Testing You

Previous research has shown that unaccompanied minors are acutely aware of other adults positioning them in a suspicious light. Narratives around problematic youth are not, as Wernesjo (2021) points out, a product of the 2016 refugee ‘crisis’ but have long existed in historical migrations to Europe. Other scholars have argued that unaccompanied minors are positioned as ‘suspicious interlopers’ within the idealised childhood, not just through age assessments but as the product of being racialised and ‘underserving’ of support (Meetoo & Rosen, 2024, p. 3). For Salim, if the ‘detective’ is there to sleuth out the ‘truth,’ then the young person being questioned becomes the ‘tested subject’ who must withstand the scrutiny. As Salim notes:

- *But the good advice*,* don’t be scared of no one. No one can do anything to you. Especially the social workers*,* yeah. They can’t do anything to you. They try their best to scare you. I know they have some tricks*,* some of them. They try to scare you. But don’t scare. If you don’t want to answer the question*,* the social workers*,* don’t worry. Just say I don’t want to answer. Sometimes they are really tricky*,* you know. They try to get you from another side. They ask you different questions but the same – it’s some question that you don’t want to answer. They ask you different. The same way*,* you know*,* the same answer you have to give. I was like*,* he was asking me some personal questions*,* my social worker. I said*,* “No*,* I don’t want to give you this answer”. He said*,* “Okay*,*” and after he asked me again. Again and again and again and again. I said*,* “No*,* bye”. And he said*,* “No*,* no*,* no*,* come”. [laugh] Salim (unaccompanied minor)*.

As noted above, Salim was one of the unaccompanied minors who saw himself as a key enactor of his own I-as-agentic-self position. He arrived at this position because of a series of negative encounters and his perceived absence of affirmative action on his behalf. Perhaps, as a result, he saw social workers’ evidence-gathering efforts as a form of trickery, or worse an attempt to “*scare you*”. Overall, this contributed to an escalation of mistrust.

Tessa, the immigration lawyer, reflected on the highly variable ways in which social workers treat the stories and disclosures made by young people. At worse, she describes how note-taking by social workers during discussions with their lawyers was a breach of confidentiality. In such contexts, the social worker’s role should be that of a corporate parent. In fact, young people may be accompanied to these appointments with a foster carer or other representative if they have one. Tessa explains an extreme situation:

- “*So*,* it varies tremendously. I mean the worst experience I ever had*,* the two brothers where the younger one came first*,* we finally got a Local Authority to agree to do an age assessment*,* and we were trying to fix an appointment for that. And*,* the social worker turned up unexpectedly outside his house and the lad wasn’t in*,* so the social worker phoned me and I said well I will find out where he is*,* and I phoned him and he wasn’t even in the city he should have been in*,* he was visiting a friend. So*,* I got back to the social worker and I said well*,* we weren’t expecting you. And he said*,* well we like to do our first visits unannounced*,* so that it doesn’t give them a chance to shave*,* pluck their eyebrows and moisturise their skin! In other words*,* they are trying to make themselves look younger than they really are. And he did in the end do an age assessment where he essentially lied. He said I can see that he has got chest hair so that is a level of growth that you wouldn’t expect in somebody under 18. And*,* when I asked the lad without him knowing why what were you wearing at that appointment*,* he had a T shirt right up to his neck*.” Tess (immigration lawyers).

For Tasneem, the support worker in a charity, the testing mechanism embedded with the system creates what she described as “*viscerally hostile*” practices at the dialogical level of self-other relationships. She argued that a focus on proving deservingness through “*gate-keeping their services and their resources*” shifts the focus to “*entitlement; it’s not a matter of kindness and generosity*”. Tasneem further notes that by extension, those who were advocating for the young people were tested and questioned as untrustworthy by extension. This was framed as an act of collusion:

- *“I don’t know how else to say it because they had actively pushed that young person to self-harming. So*,* when we are telling you time and time again what’s causing it and they ask that. They upped their ante on all the causes of his suicidal ideation and self-harming and then when we tried to advocate*,* which is what we’re supposed to do*,* personal attacks on us. So*,* it’s just like*,* “You’re conducting yourself poorly”*,* or*,* “This is something that you’ve done that’s wrong”*,* or*,* “You’ve lied to children”. I mean it’s been quite relentless*,* and it shouldn’t have to be like that.”* (Tasneem, support worker in charity).

Previous research has shown that professionals’ responses to managing to the challenges of working with unaccompanied minors was varied. Some discussed the young people’s legitimacy in negative and sometimes emotionally distant ways. Others were deeply compassionate and found ways to overtly or covertly disrupt the system(Hadwin & Singh, 2025; Humphris & Sigona, 2019).

## Concluding Thoughts

From the outset of our empirical work, we were deeply interested in examining how contradictions in a system, designed to protect children because of their child status, simultaneously created barriers to their asylum provision because of their immigration status. In turn, this shaped the quality of their care. In this paper we have been asking: (1) Can a dialogical theorising provide deeper insights into what ‘crisis’ might look like at the microsocial level? (2) How might macrosocial elements influence what happens self-other dynamics between unaccompanied minors and adults? (3) How might relationships that should be grounded in care become a sight of tension and misalignment and can this be considered a form of crisis? (4) Can our previous work examining professional adults’ used of generalised concepts of child development and childhood be used to inform a deeper understanding of self-other dynamics and in particular, where misalignments occur? We drew on sociocultural approaches to dialogical theorising to help illustrate how crisis is made through meaning-making in the everyday dialogical interplay between the self (I-positions) and ‘they-other’ relationships. This meaning-making at the dialogical level is also influenced by wider macrosocial processes such as immigration regimes, frameworks and practices.

Our first contribution to the field lies in applying sociocultural psychological approaches to dialogical self-other relationships, the concepts of recognition and misrecognition and dialogical approaches to trust and mistrust. They have helped to make sense of these microsocial processes of the ‘knower’ and the ‘not-knower’. Or rather, who knows what about whom? We have argued that the crisis is made in the everyday sense-making in self-other misalignments between young people and adult professionals, which leads to forms of identity misrecognition and eventually, a breakdown in trust. For example, the identity position as **‘I’-as-agentic-self** was seen in a positive light by those unaccompanied minors who felt they had built their capacity for agency as part of their welfare and asylum journey. Even if their perceived sense of agency often arose in response to systematic and professional failure to provide affirmative action for their needs. Conversely, this identity could be misrecognised by adults who interpreted such agency as evidence of the young people being **savvy users of the system**. These misalignments in self-other relationships have significant practical implications for professionals’ ability to view young people through a lens of care and compassion(Sirriyeh, 2018). Once established, young people may become stuck in the taboo I-position (Valsiner & Cabell, 2012). Combined with high levels of bureaucracy, this can lead to waning levels of trust (Marková et al., 2008) or as Salim put it, caught in a “*game*”. For someone like Joshua, who did not describe an **‘I’-as-agentic-self**, identity, the result was feeling of being both physically and symbolically imprisoned.

Our second contribution concerns the dialogical loop that emerges between unaccompanied minors and adult professionals. Through this analytic lens, both groups participate in what Cantat et al. (2023, p. 3) describe as ‘assemblage’s of events, discourses/representations and practices,’ that promote a sense of misrecognition, fuel mistrust, and exacerbate misalignments in self-other identities. For the young people, the repetitive questioning by adults, where previous conversations had seemingly been “*forgotten*,” led to more questions, and created a perceived dialogical loop of **‘They’ are testing you**. For adults, particularly social workers, these conversations served as a necessary rapport-building exercise but in some cases, were used to “*catch-out*” young people in a lie. While unaccompanied minors sought action for change, adults were often engaged in procedural or relational dialogue. In the absence of concrete action, young people took control themselves.

In this regard, the migration and asylum system inadvertently becomes a training ground for agentic capacity-building. Yet simultaneously, also sustained a dialogical loop in which young people are positioned as ‘savvy’ knowers of the system. State social workers explicitly told us that they were able to mine personal narratives, wider connections (e.g. foster carer narratives) and social media for “*intelligence*”, which contributes to the **‘I’ as detective** into the **‘They’ are testing you**, loop. It is useful to return to the conceptual notion of mistrust and how this plays out in a dialogical analysis across different interviews. Our paper takes the previous excellent work on escalations of trust in dialogical turn-taking a step further by arguing that mistrust can be analysed beyond a singular conversation through dialogical descriptions and interactions across multiple experiences. We suggest that mistrust develops over time as a cumulative process – a collation of interactions.

Thirdly, Heidbrink (2020) argues that the hierarchy of vulnerability framing of unaccompanied minors, presumes vulnerability is inherent in the status of being a minor, and portrays children as passive, dependent and victimised. Within this framing, vulnerability is assumed to lessen with age, transferring power to expert professionals whilst reducing young people’s perceived capacity for agency. The vulnerability hierarchy individualises this process, rather than questioning the contribution made by wider macrosocial dynamics. From a dialogical perspective, we suggest that the intersection between those macro and micro dynamics are part of what creates the dialogical loop. The dialogical dynamics are constructed at the interpersonal level in self-other interactions but always shaped by broader social and institutional assemblages. Although the migration system can act as a training ground for agentic capacity-building, such agency is rarely recognised positively. Instead of viewing it as part of a learning process in the transition to adulthood, the system’s emphasis on vulnerability penalises young people for not remaining passive recipients of adult intervention. In other words, the system creates the problem, young people respond by becoming agentic or feeling stuck, but their agentic response does not fit the expected narrative of the vulnerable minor. Consequently, the agentic child is reframed as savvy, prompting social workers to seek more “evidence,” which in turn reinforces young people’s feelings of being constantly tested. Thus, the dialogical loop of self–other misalignment persists.

For practitioners who must collect information to support their care work, recognising their own reflexive position within the loop of misrecognition and mistrust is crucial. Awareness of the dialogical loop offers an opportunity to break it. The burueaucratic demands of the job, particularly for social workers and personal advisors in England, can easily become the dominant focus of professional practice. Our data show that while unaccompianed minors understand the necessity of such procedures, they place equal importance on action-oriented change. Relationships with professionals outside the state sector often appear smoother, perhaps because they are more practically focused. Adults could therefore reframe young people’s knowledge of the system and their growing independence, not as grounds for suspicion, but as evidence of skill development and a form of informal learning that occur through lived experience of the transitions to adulthood. In a previous work, we have also argued that practitioners could benefit from reflexive training that draws on critical-developmental theorising to challenge how generalised views of children’s development can be unpacked to help avoid negative views about children’s agentic actions(Crafter & Ott, online first). In doing so, this could be a step towards reducing misrecognition and mistrust.

## Data Availability

The anonymised datasets generated by this research are currently available under restricted access in the UK Data Service repository https://doi.org/10.5255/UKDA-SN-856744.

## Footnotes

1 European Parliament and Council of the European Union. (2013). Regulation (EU) No 604/2013. Official Journal of the European Union, L 180, 31–59. https://eur-lex.europa.eu/legal-content/EN/TXT/?uri=CELEX%3A32013R0604.
